# Supplementary material for: Neuroinflammation in an Optimized Model of Lysophosphatidic Acid (LPA)-Induced Post-hemorrhagic Hydrocephalus
Source: Neurochem Res. 2025 Sep 19;50(5):305. doi: 10.1007/s11064-025-04542-4 (PMC12449426; doi:10.1007/s11064-025-04542-4)
Supplement: Supplementary file 1 — Supplementary Material 1 [file 11064_2025_4542_MOESM1_ESM.docx]

**Online Resources 2, 3, and 6**

**Neuroinflammation in an optimized model of lysophosphatidic acid (LPA)-induced post-hemorrhagic hydrocephalus**


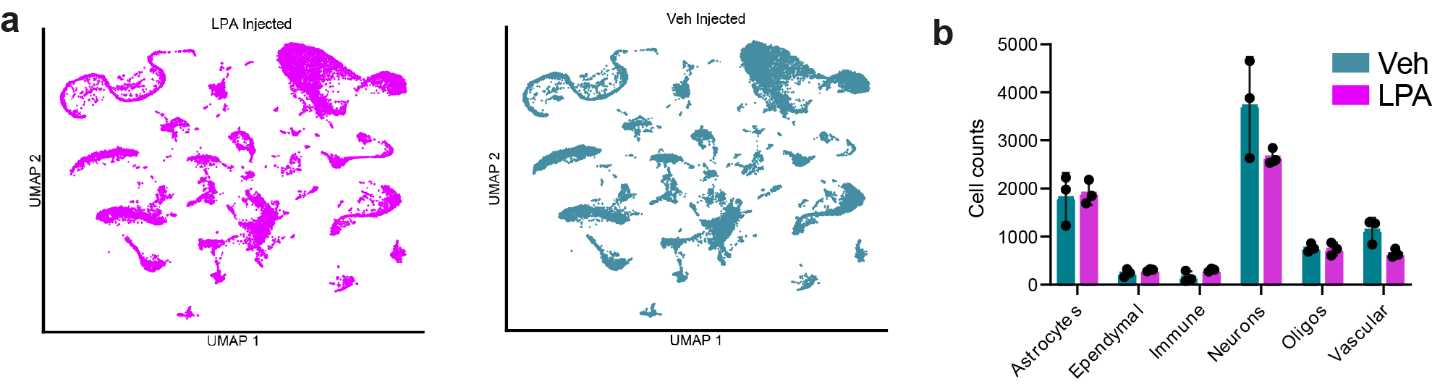


Online Resource 2. (**a**) UMAPs of LPA-injected (left) and vehicle (Veh)-injected (right) nuclei separated by treatment group. (**b**) Number of nuclei profiled for each individual sample grouped by vehicle or LPA treatment. Bars represent mean ± s.d. and circles represent values from individual mice


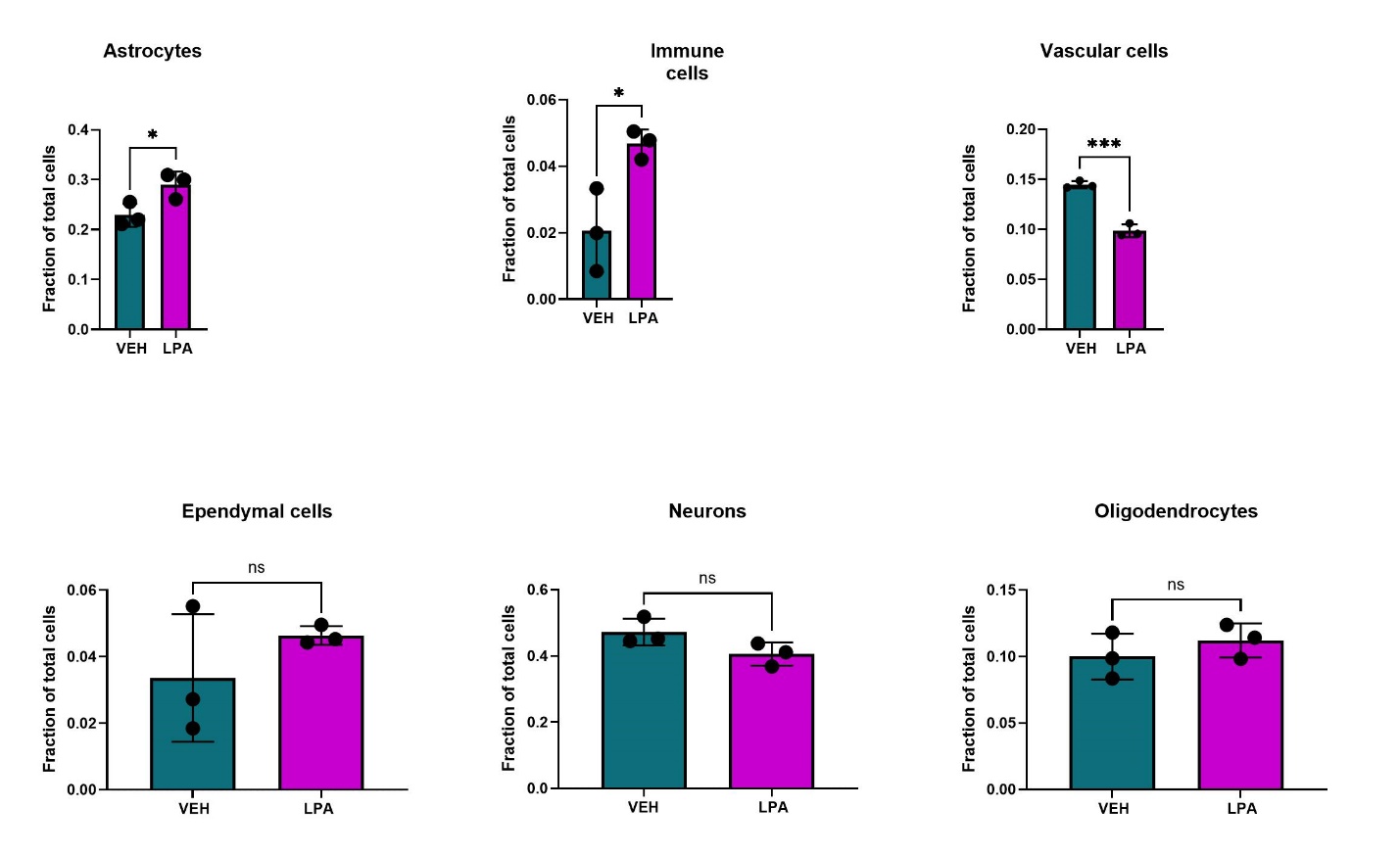


Online Resource 3. Proportions of cell types from snRNA-seq data. Bars represent mean ± s.d. and circles represent values from individual mice.


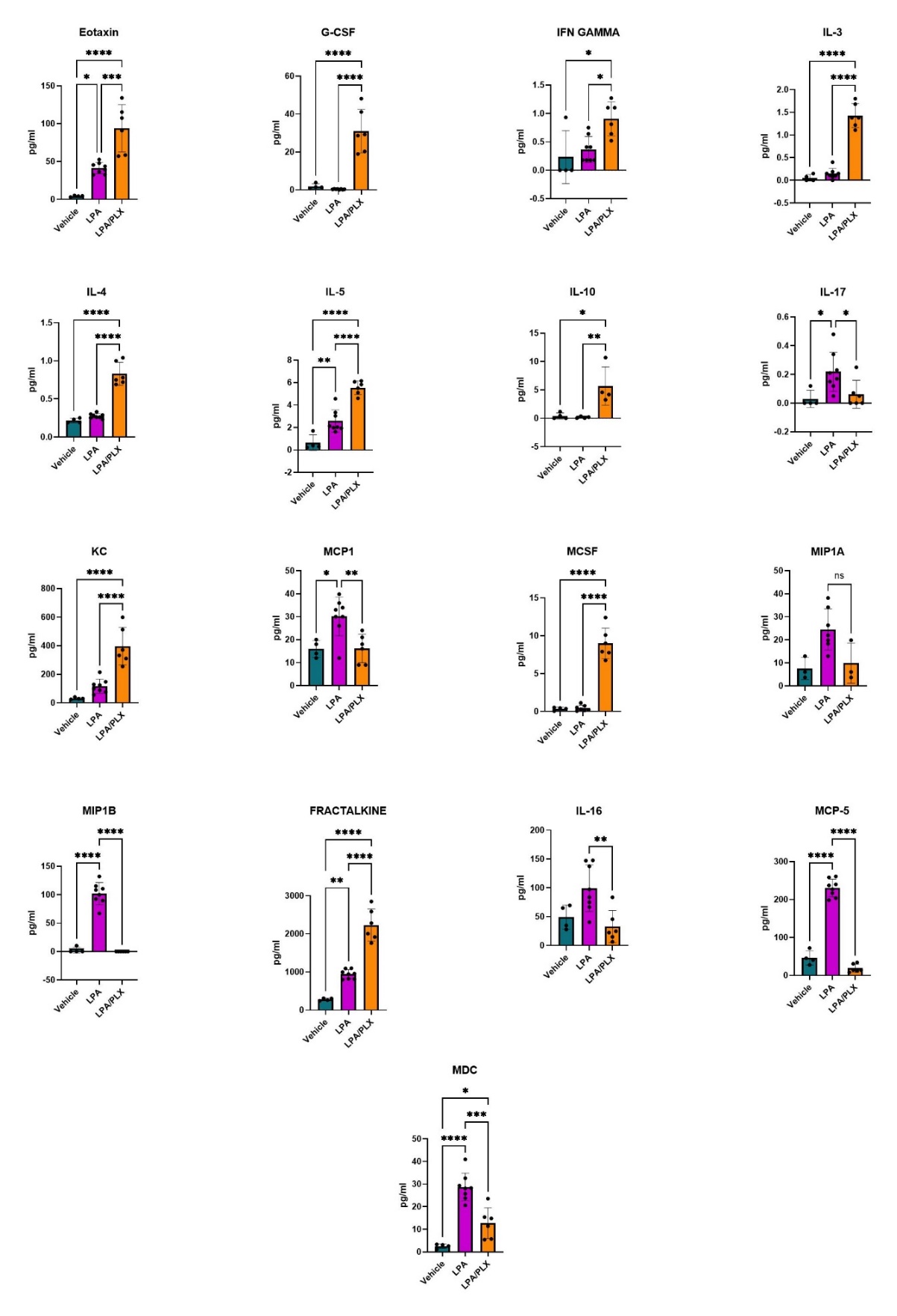


Online Resource 6. Quantification of chemokines and cytokines in CSF of vehicle-, LPA-, and LPA/PLX 3397-injected animals as measured by the mouse cytokine/chemokine 45-Plex Discovery Assay (MD45). LPA values are from individual animals, vehicle values are from samples created by pooling CSF from three individual mice. Vehicle-injected, *n* = 4; LPA-injected, *n* = 5. *p*-values from ANOVA followed by post-test with Tukey’s correction for multiple comparisons: **p* < 0.05, ***p* < 0.01, ****p* < 0.001, *****p* < 0.0001.
